# Requirement of Cholesterol for Calcium‐Dependent Vesicle Fusion by Strengthening Synaptotagmin‐1‐Induced Membrane Bending

**DOI:** 10.1002/advs.202206823

**Published:** 2023-04-14

**Authors:** Houda Yasmine Ali Moussa, Kyung Chul Shin, Janarthanan Ponraj, Soo Jin Kim, Je‐Kyung Ryu, Said Mansour, Yongsoo Park

**Affiliations:** ^1^ Neurological Disorders Research Center Qatar Biomedical Research Institute (QBRI) Hamad Bin Khalifa University (HBKU) Qatar Foundation Doha Qatar; ^2^ HBKU Core Labs Hamad Bin Khalifa University (HBKU) Doha Qatar; ^3^ Division of Molecular and Life Sciences Pohang University of Science and Technology Pohang 790‐784 Republic of Korea; ^4^ Department of Physics & Astronomy Seoul National University. 1 Gwanak‐ro Gwanak‐gu Seoul 08826 South Korea; ^5^ College of Health & Life Sciences (CHLS) Hamad Bin Khalifa University (HBKU) Qatar Foundation Doha Qatar

**Keywords:** cholesterol, curvature, SNARE, synaptotagmin‐1, vesicle fusion

## Abstract

Cholesterol is essential for neuronal activity and function. Cholesterol depletion in the plasma membrane impairs synaptic transmission. However, the molecular mechanisms by which cholesterol deficiency leads to defects in vesicle fusion remain poorly understood. Here, it is shown that cholesterol is required for Ca^2+^‐dependent native vesicle fusion using the in vitro reconstitution of fusion and amperometry to monitor exocytosis in chromaffin cells. Purified native vesicles are crucial for the reconstitution of physiological Ca^2+^‐dependent fusion, because vesicle‐mimicking liposomes fail to reproduce the cholesterol effect. Intriguingly, cholesterol has no effect on the membrane binding of synaptotagmin‐1, a Ca^2+^ sensor for ultrafast fusion. Cholesterol strengthens local membrane deformation and bending induced by synaptotagmin‐1, thereby lowering the energy barrier for Ca^2+^‐dependent fusion to occur. The data provide evidence that cholesterol depletion abolishes Ca^2+^‐dependent vesicle fusion by disrupting synaptotagmin‐1‐induced membrane bending, and suggests that cholesterol is an essential lipid regulator for Ca^2+^‐dependent fusion.

## Graphical abstract

A schematic diagram summarizing synaptotagmin‐1‐induced membrane bending for Ca^2+^‐dependent vesicle fusion. PIP_2_ and cholesterol are critical for Ca^2+^‐dependent vesicle fusion. Synaptotagmin‐1 mediates Ca^2+^‐dependent vesicle fusion by i) membrane insertion and ii) interacting with PIP_2_ through the polybasic patch, which induces the *trans*‐interaction of synaptotagmin‐1. Cholesterol strengthens the plasma membrane deformation and bending caused by synaptotagmin‐1 insertion.

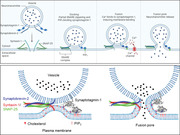

## 1 Introduction

Cholesterol, a major component in cell membrane bilayers, is essential for membrane structure and fluidity. The brain is the most cholesterol‐enriched organ and a human brain contains about 20–25% of the body's cholesterol;^[^ ^1^ ^]^ this high density suggests that cholesterol has a critical function in the brain. Age‐related cholesterol reduction in the frontal and temporal cortices^[^ ^2^ ^]^ results in loss of synaptic contacts, changes in neuronal morphology, and reduced synaptic plasticity.^[^ ^3^ ^]^ Cholesterol is associated with neurogenesis, neurodevelopment, and synaptogenesis.^[^ ^4^ ^]^ Age‐related cholesterol deficiency in the plasma membrane leads to deficits in synaptic plasticity in mouse hippocampal neurons,^[^ ^5^ ^]^ and cholesterol depletion by methyl‐*β*‐cyclodextrin (M*β*CD) from the plasma membrane impairs neurotransmission and neuronal activity, thereby leading to synapse degeneration.^[^ ^6^ ^]^

The plasma membrane contains ≈80% of total cellular cholesterol.^[^ ^7^ ^]^ Cholesterol is capable of clustering syntaxin‐1A^[^ ^8^ ^]^ so that soluble *N*‐ethylmaleimide‐sensitive factor attachment protein receptor (SNARE) proteins become concentrated in cholesterol‐enriched domains in the plasma membrane.^[^ ^9^ ^]^ Depletion and reduction of cholesterol level in the plasma membrane inhibit Ca^2+^‐dependent exocytosis of large dense‐core vesicles (LDCVs)^[^ ^10^ ^]^ and cortical secretory vesicles from sea urchins,^[^ ^11^ ^]^ as well as synaptic vesicles (SVs) in hippocampal neurons,^[^ ^6^, ^12^ ^]^ cortical synaptosomes,^[^ ^13^ ^]^ ribbon synapses,^[^ ^14^ ^]^ and motor nerve terminals.^[^ ^15^ ^]^ However, the molecular mechanisms by which cholesterol deficiency disrupts synaptic transmission and induces neurodegeneration remain elusive.

Exocytosis is the process of neurotransmitter release through merging two lipid bilayers, which is drived by SNARE proteins.^[^ ^16^ ^]^ Neuronal SNARE proteins consist of Q‐SNARE in the plasma membrane (syntaxin‐1 and SNAP‐25) and R‐SNARE in the vesicle membrane (synaptobrevin‐2 or vesicle‐associated membrane protein‐2 (VAMP‐2)).^[^ ^16a^ ^]^ Synaptotagmin‐1 is responsible for ultrafast Ca^2+^‐dependent exocytosis. Synaptotagmin‐1 contains two tandem C2‐domains that coordinate Ca^2+^ binding; the Ca^2+^‐bound C2 domain can be inserted into negatively charged anionic phospholipids by electrostatic interaction.^[^ ^16b^ ^]^ In spite of intense investigation of synaptotagmin‐1, the molecular mechanisms by which synaptotagmin‐1 mediates Ca^2+^‐dependent vesicle are still under debate.^[^ ^17^ ^]^

Cholesterol regulates fusion pore formation during LDCV exocytosis in chromaffin cells.^[^ ^10^, ^18^ ^]^ Amperometry is an electrochemical technique that recognizes the release of catecholamines‐containing LDCVs and detects even a single vesicle fusion event in real‐time.^[^ ^19^ ^]^ Amperometry data show that cholesterol controls fusion pore formation during exocytosis^[^ ^10^, ^18^ ^]^ and constrains the narrow semi‐stable fusion pore.^[^ ^18b^ ^]^ Given that cholesterol depletion affects various cellular signaling pathways and the activity of ion channels, it is challenging to investigate the specific role of cholesterol on vesicle fusion. To overcome this limitation and address the mechanisms of cholesterol for vesicle fusion before fusion pore formation, we applied the reconstitution system of vesicle fusion by using purified native vesicles; that is, LDCVs and SVs. This reconstitution of native vesicle fusion enables us to come up with novel mechanisms for how cholesterol regulates vesicle fusion before fusion pore occurs.

Here, we propose that cholesterol is required for Ca^2+^‐dependent vesicle fusion by using amperometry and the in vitro reconstitution of vesicle fusion. Importantly, vesicle‐mimicking liposomes (V‐liposomes) fail to reproduce the cholesterol effect, indicating that purified native vesicles, that is, LDCVs and SVs, are crucial for the complete reconstitution of physiological Ca^2+^‐dependent fusion. Membrane binding of synaptotagmin‐1 occurs regardless of cholesterol. Transmission electron microscope (TEM) reveals that cholesterol strengthens membrane bending and curvature induced by the insertion of synaptotagmin‐1, and therefore the membrane bending energy can lower the energy barrier for Ca^2+^‐dependent fusion.

## 2 Results

### 2.1 Cholesterol Depletion Causes Deficits in Exocytosis

**Figure 1:**
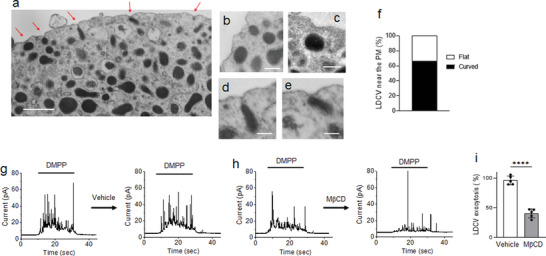
Cholesterol depletion inhibits LDCV exocytosis in chromaffin cells. a) Transmission electron microscope (TEM) images of chromaffin cells showing invagination of the plasma membrane (PM) into LDCVs. Scale, 500 nm. b–e) Magnified TEM images of LDCVs. Scale, 200 nm. f) LDCVs close to the PM less than 100 nm distance between LDCV and PM were counted; LDCVs near either flat or curved PM are presented as a percentage (a total of 36 LDCVs proximal to the PM from three independent experiments). g–i) LDCV exocytosis in chromaffin cells measured by amperometry. M*β*CD (10 mm, 2 h) reduced LDCV exocytosis of chromaffin cells (h). g,h) Shown are typical amperometric traces upon DMPP stimulations finally activating voltage‐gated calcium channels for 20 s. Chromaffin cells were treated with either vehicle (g) or M*β*CD (h) for 2 h after the first DMPP stimulation. i) Amperometric current generated by repetitive stimulation was individually integrated. Relative LDCV exocytosis is presented as a percentage of the first DMPP‐induced total catecholamine release. Data are means ± SD from five independent experiments. Unpaired two‐tailed *t*‐test; ****, *p* < 0.0001.

We investigated whether local membrane curvature is observable in vivo in primary chromaffin cells. Using electron microscopy (**Figure** **1**a–f), we observed the invagination of the plasma membrane into LDCVs, suggesting that the plasma membrane could be already curved before Ca^2+^‐dependent fusion happens. Cholesterol induces spontaneous membrane curvature and bending,^[^ ^20^ ^]^ thereby promoting highly curved membrane intermediate structures in membrane fusion.^[^ ^21^ ^]^ Moreover, high‐curvature membrane domains, such as caveolae, are heavily enriched with cholesterol, which is essential for membrane invaginations.^[^ ^20^, ^22^ ^]^ Cholesterol depletion by M*β*CD or nystatin disrupts membrane invaginations and leads to a flattening of the curved caveolae membrane structure.^[^ ^23^ ^]^

Given that the curved and invaginated membranes are enriched with cholesterol^[^ ^20^, ^23^ ^]^ and ≈67% of vesicles are proximal to the invaginated plasma membranes (Figure 1f), the membrane invagination to LDCVs we observed is likely to be cholesterol‐enriched regions, where SNARE proteins are present^[^ ^24^ ^]^ (Figure 1a–e). To test the cholesterol effect on vesicle fusion, we used amperometry to monitor LDCV exocytosis in real‐time. Indeed, treatment with M*β*CD, which depletes cholesterol from the plasma membrane, inhibited Ca^2+^‐dependent LDCV fusion by ≈60% (Figure 1g–i).

### 2.2 Cholesterol is Required for Ca^2+^‐Dependent Vesicle Fusion

**Figure 2:**
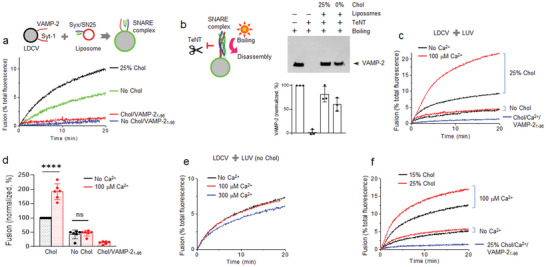
Cholesterol is required for Ca^2+^‐triggered vesicle fusion. a) In vitro reconstitution of a large dense‐core vesicle (LDCV) fusion using a lipid‐mixing assay. Purified LDCVs were incubated with PM‐liposomes that incorporate the stabilized Q‐SNARE complex of syntaxin‐1A/SNAP‐25A (Syx/SN25) in a 1:1 molar ratio (Experimental Section). Cholesterol (Chol) either 25% or 0% was included in PM‐liposomes. For clarity, only endogenous VAMP‐2 and synaptotagmin‐1 of native LDCVs are shown and full fusion is presented. Similar results were obtained from six independent trials (*n* = 6). Preincubation of PM‐liposomes with VAMP‐2_1‐96_, the cytoplasmic domain of VAMP‐2 (aa 1–96), specifically disrupted SNARE‐dependent vesicle fusion. b) Formation of the ternary SNARE complex after vesicle fusion in the presence or absence of cholesterol in PM‐liposomes. LDCVs were incubated with liposomes for 20 min without Ca^2+^, then treated with Tetanus neurotoxin (TeNT). TeNT‐resistant VAMP‐2 indicates the ternary SNARE complex formation in SDS‐PAGE (Experimental Section). Boiling at 95 °C disrupts the ternary SNARE complex so that VAMP‐2 migrates to its size. Data are mean ± SD from three independent experiments (*n* = 3). c,d) Dependence on cholesterol for Ca^2+^‐dependent vesicle fusion. The addition of 100 µm free Ca^2+^ provoked the fusion of LDCV with PM‐liposomes. Fusion is presented as a percentage of basal fusion with Chol‐containing PM‐liposomes in the absence of Ca^2+^. Data are mean ± SD (*n* = 6 independent experiments). One‐way ANOVA test with Bonferroni correction was used; ****, *p* < 0.0001; ns, not significant. e) Free Ca^2+^ was increased to 300 µm when LDCVs fused with PM‐liposomes (0% Chol). f) Liposomes contained either 15% or 25% of Chol. Similar results in (e,f) were obtained from four independent trials. Lipid composition of PM‐liposomes: 45% PC, 15% PE (including labeled PE), 10% PS, 25% Chol, 4% PI, and 1% PI(4,5)P_2_. When Chol was reduced, PC contents were adjusted accordingly. Physiological ionic strength and 1 mm MgCl_2_/3 mm ATP were used in all experiments.

To further study the molecular mechanisms by which cholesterol deficiency affects vesicle fusion, we applied a reconstitution system of vesicle fusion by using purified native vesicles, such as LDCVs and SVs, as reported previously.^[^ ^25^ ^]^ The plasma membrane‐mimicking liposomes (PM‐liposomes) contain the stabilized Q‐SNARE complex (syntaxin‐1A and SNAP‐25A in a 1:1 molar ratio)^[^ ^26^ ^]^ (Experimental Section). We first tested the effect of cholesterol on basal fusion without Ca^2+^. LDCV fusion with PM‐liposomes was readily observed (**Figure** **2**a), but the absence of cholesterol in PM‐liposomes reduced LDCV fusion by ≈50% (Figure 2a,d); PM‐liposomes contain either 25% or 0% cholesterol (Chol). As a control, the pre‐incubation of VAMP‐2_1‐96_, the soluble cytoplasmic region of VAMP‐2, blocked vesicle fusion, supporting SNARE‐dependent vesicle fusion.

The efficiency of the SNARE assembly and SNARE complex formation was examined using the light chain of tetanus toxin (TeNT), a protease specific for free VAMP‐2; that is, VAMP‐2 in the assembled SNARE complexes is resistant to the cleavage by TeNT so that TeNT‐resistant VAMP‐2 represents the ternary SNARE complex formation.^[^ ^25a^ ^]^ After LDCV fusion with PM‐liposomes as a fusion assay in Figure 2a, TeNT was added to cleave free VAMP‐2, and we quantified VAMP‐2 engaged in the SNARE complex formation. The ternary SNARE complex formation was observed both in the presence (25% Chol) and absence of cholesterol (0% Chol) in PM‐liposomes (Figure 2b), suggesting that SNARE proteins are assembled regardless of cholesterol.

Then we investigated the cholesterol effect on Ca^2+^‐dependent fusion. Although cholesterol controls Ca^2+^‐dependent neurotransmission and exocytosis in diverse cell types,^[^ ^6^, ^10^, ^11^, ^12^, ^13^, ^14^, ^15^ ^]^ the step at which vesicle fusion is impaired has not been determined. We used native vesicles for the complete reconstitution of vesicle fusion to reproduce physiological Ca^2+^‐dependent vesicle fusion, correlating with the in vivo data in a physiological ionic environment.^[^ ^25^ ^]^ Addition of 100 µm free Ca^2+^ accelerated vesicle fusion in the presence of Mg^2+^/ATP (Figure 2c,d), where synaptotagmin‐1 interacts only with PIP_2_‐containing membranes, but not SNARE proteins.^[^ ^25a,b^ ^]^ Surprisingly, Ca^2+^‐evoked LDCV fusion was completely abolished when cholesterol in PM‐liposomes was absent (Figure 2c,d). The Q‐SNARE proteins were incorporated in PM‐liposomes independently of cholesterol (Figure S1, Supporting Information). We also confirmed that Ca^2+^‐dependent LDCV fusion did not occur without cholesterol (0% Chol) in PM‐liposomes when the full‐length syntaxin‐1A and SNAP‐25A binary acceptor complex (see Experimental Section) was included (Figure S2, Supporting Information). Different concentrations of free Ca^2+^ failed to increase vesicle fusion when PM‐liposomes contained no cholesterol (Figure 2e), and cholesterol accelerated Ca^2+^‐dependent LDCV fusion in a dose‐dependent manner (Figure 2f). Altogether, our results confirm that cholesterol is required for Ca^2+^‐dependent vesicle fusion and this in vitro reconstitution reproduces the physiological roles of cholesterol on vesicle fusion and exocytosis.

### 2.3 Cholesterol is not Required for Liposome–Liposome Fusion

**Figure 3:**
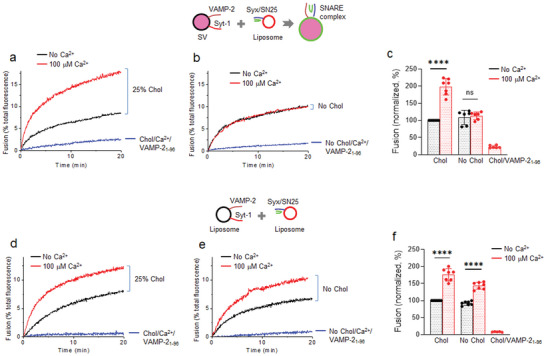
Cholesterol is not required for liposome–liposome fusion. a–c) Synaptic vesicle (SV) fusion with PM‐liposomes that contain either 25% (a,c) or 0% (b,c) Chol. Only endogenous VAMP‐2 and synaptotagmin‐1 of native SVs are shown and full fusion is presented for clarity. d–f) Instead of native LDCVs or SVs, V‐liposomes incorporating the full‐length synaptotagmin‐1 and VAMP‐2 were incubated with PM‐liposomes that contained either 25% (d) or 0% (e) Chol. Lipid composition of PM‐liposomes is described in Figure 2. Lipid composition of V‐liposomes: 55% PC, 20% PE, 15% PS, and 10% Chol. c,f) Fusion is presented as a percentage of basal fusion with Chol‐containing PM‐liposomes in the absence of Ca^2+^. Data in (c) and (f) are mean ± SD from six to seven independent experiments (*n* = ≈6–7). One‐way ANOVA test with Bonferroni correction was used; ****, *p* < 0.0001; ns, not significant.

We further tested SVs purified from mice brains to determine whether cholesterol is essential for Ca^2+^‐dependent SV fusion. Indeed, Ca^2+^ failed to accelerate SV fusion in the absence of cholesterol in PM‐liposomes (0% Chol) (**Figure** **3**a–c); this result is consistent with the cholesterol requirement for Ca^2+^‐dependent LDCV fusion (Figure 2d), however, Ca^2+^‐independent basal SV fusion was not affected (Figure 3c). A unique and major advantage of native vesicles for a fusion assay is that they maintain the native lipid and protein diversity, as well as the structural integrity of vesicles to mimic endogenous and physiological vesicle fusion. Instead of purified native vesicles, vesicle‐mimicking liposomes (V‐liposomes) have been used for a fusion assay to study the molecular mechanisms. We, therefore, tested V‐liposomes that incorporate full‐length VAMP‐2 and synaptotagmin‐1 to examine whether the dependence on cholesterol for Ca^2+^‐dependent fusion can be reproduced (Figure 3d–f). Intriguingly, Ca^2+^‐dependent liposome fusion was slightly reduced but still significantly observable even in the absence of cholesterol in PM‐liposomes (Figure 3d–f). These results indicate that cholesterol is required for Ca^2+^‐dependent fusion of native vesicles, that is, LDCV and SV, but not for liposome–liposome fusion.

### 2.4 Membrane Binding of Synaptotagmin‐1 Independently of Cholesterol

**Figure 4:**
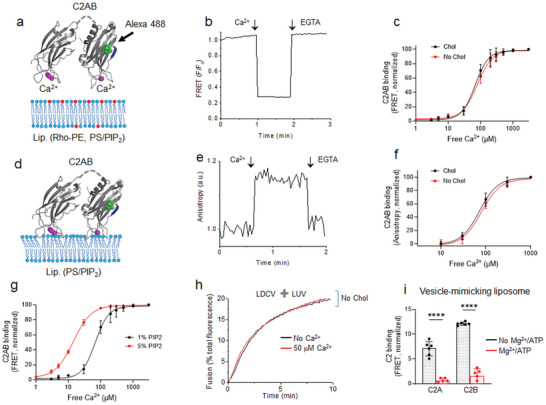
Membrane binding of the C2AB domain of synaptotagmin‐1 in the presence or absence of cholesterol. a–c) Membrane binding of the C2AB domain of synaptotagmin‐1 was monitored using FRET in which the C2AB domain (Syt‐1_97‐421_) was labeled with Alexa Fluor 488 at S342C (green dots) as a donor, and PM‐liposomes (Lip.) were labeled with Rhodamine (Rho)‐PE (red) as an acceptor (Experimental Section). PM‐liposomes (protein‐free) contained either 25% or 0% Chol. c) C2AB binding is presented as a percentage of maximum C2AB binding induced by 1 mm free Ca^2+^. Data are mean ± SD from ≈6–7 independent experiments. d–f) Membrane insertion of the C2AB domain to PM‐liposomes (protein‐free; lipid composition as in (a–c) without Rho‐PE) was monitored using fluorescence anisotropy. f) Dose‐response curve of Ca^2+^‐dependent C2AB binding to PM‐liposomes that contain either 25% or 0% Chol. Data are mean ± SD (*n* = 6 independent experiments). Bonferroni‐corrected two‐way ANOVA test was used for (c) and (f); not significant between Chol and No Chol. g) FRET was conducted to monitor C2AB binding to liposomes as in (a–c). PIP_2_ was incorporated in PM‐liposomes (25% cholesterol included, protein‐free). High PIP_2_ concentration increased Ca^2+^ sensitivity for C2AB binding to membranes. Data are mean ± SD (*n* = ≈6–7 independent experiments). h) LDCV fusion with PM‐liposomes that contain 5% PIP_2_ concentration and no Chol. Similar results were obtained from four independent trials. i) C2AB binding to V‐liposomes was monitored using a tryptophan‐dansyl FRET pair. Neither the C2A nor C2B domain binds to V‐liposomes (no PIP_2_) by 1 mm Ca^2+^ in the presence of 1 mm MgCl_2_/3 mm ATP. Data are mean ± SD (*n* = 6 independent experiments). Unpaired two‐tailed *t*‐test; ****, *p* < 0.0001.

Cholesterol in PM‐liposomes is indispensable for Ca^2+^‐dependent vesicle fusion, so we investigated whether the binding of synaptotagmin‐1 to the PIP_2_‐containing membrane is affected by cholesterol. The C2AB domain of synaptotagmin‐1 (Syt_97‐421_) was labeled with Alexa Fluor 488 at S342C as a donor, and PM‐liposomes (Lip., protein‐free) were labeled with Rhodamine (Rho)‐PE as an acceptor (Experimental Section). The C2AB binding to liposomes was monitored by using fluorescence resonance energy transfer (FRET) between the C2AB domain (Alexa Fluor 488) and Rhodamine‐labeled PM‐liposomes (**Figure** **4**a). The C2AB domain of synaptotagmin‐1 could bind to both cholesterol‐containing and cholesterol‐free liposomes in response to Ca^2+^, and the Ca^2+^ titration for C2AB binding to PM‐liposomes (0% Chol) was comparable to that of 25% Chol‐containing PM‐liposomes; this result demonstrates Ca^2+^‐dependent C2AB binding to anionic phospholipids regardless of cholesterol in PM‐liposomes (Figure 4a–c). PM‐liposomes contain anionic phospholipids, including 10% PS, 4% PI, and 1% PIP_2_, which provide complete coordination sites for the Ca^2+^‐bound C2AB domain to interact with the membranes (Figure 4a–c).

To further assess membrane insertion of the C2AB domain, a fluorescence anisotropy measurement was conducted to monitor the rotational mobility of the C2AB domain, labeled with Alexa Fluor 488 at 342C; PM‐liposomes are label‐free and protein‐free (Figure 4d). Membrane insertion of the C2AB domain results in the increase of fluorescence anisotropy due to a reduction in the rotational mobility of the membrane‐bound C2AB domain (Figure 4e). Ca^2+^ titration for membrane binding of the C2AB domain showed no difference between the two sets of PM‐liposomes (0% vs 25% Chol) (Figure 4d–f). The increases in PIP_2_ concentration in PM‐liposomes shifted Ca^2+^ titration curves to the left, indicating an increase in the Ca^2+^‐sensitivity of C2AB membrane binding (Figure 4g and Figure S3, Supporting Information); EC_50_: 68.1 µm Ca^2+^ for 1% PIP_2_ and 14.3 µm Ca^2+^ for 5% PIP_2_. High PIP_2_ concentration increases Ca^2+^ sensitivity for vesicle fusion;^[^ ^25^, ^27^ ^]^ this interaction implies that an increase in the magnitude of the negative electrostatic potential in the plasma membranes increases the attraction of Ca^2+^‐bound synaptotagmin‐1. Even with 5% PIP_2_ in PM‐liposomes, Ca^2+^ still failed to increase LDCV fusion without cholesterol (Figure 4h).

We have previously reported the membrane binding of the C2AB domain in a physiological ionic environment,^[^ ^25a,b^ ^]^ showing that the C2AB domain can only bind to the PIP_2_‐containing plasma membrane, not to the SNARE complex. Then, we further validated if either the C2A or C2B domain still binds to the vesicle membrane at physiological ionic strength. Indeed, the C2A and C2B domains bind to V‐liposomes (0% PIP_2_, 15% PS included), but this interaction was completely disrupted at physiological ionic strength due to the electrostatic effect of Mg^2+^ and ATP (Figure 4i).^[^ ^25^, ^27^ ^]^ Both the C2A and C2B domains bind to the plasma membrane to drive vesicle fusion in a physiological ionic environment,^[^ ^25^, ^27^ ^]^ proposing that membrane binding of both the C2A and C2B domains have a cooperative and synergistic effect on membrane deformation. Taken together, C2AB binding into the plasma membrane directly provides the driving force to lower the energy barrier for Ca^2+^‐triggered vesicle fusion to occur. Cholesterol is only required for Ca^2+^‐dependent vesicle fusion but is not essential for Ca^2+^‐dependent membrane binding of the C2AB domain.

### 2.5 Membrane Curvature is Critical for Ca^2+^‐Dependent Fusion

Despite the complete inhibition of Ca^2+^‐dependent vesicle fusion (Figures 2 and 3a,b), membrane binding of the C2AB domain was still observable in the absence of cholesterol in PM‐liposomes (Figure 4); this observation strongly suggests that the downstream membrane binding of synaptotagmin‐1 is disrupted. Hydrophobic residues in the Ca^2+^‐binding loops of synaptotagmin‐1 penetrate the inner leaflet of the plasma membrane,^[^ ^28^ ^]^ and probably lead to local membrane bending and deformation.^[^ ^29^ ^]^ This local membrane deformation might accelerate vesicle fusion by lowering the energy barrier.^[^ ^30^ ^]^ Cholesterol also regulates local membrane bending and deformation,^[^ ^31^ ^]^ so in the next experiments, we examined whether cholesterol has a critical function in membrane bending to trigger Ca^2+^‐dependent vesicle fusion.

**Figure 5:**
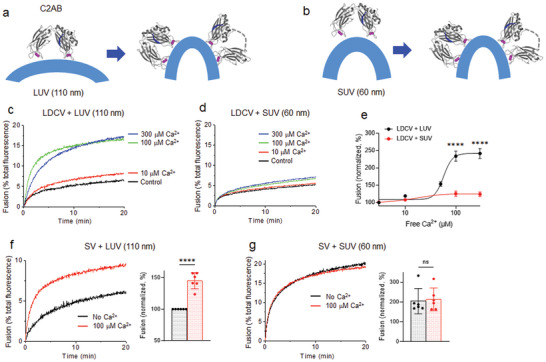
High curvature strain of membrane increases basal fusion but decreases Ca^2+^‐triggered fusion. a,b) Schematic diagram showing membrane bending by binding of the C2AB domain. Large unilamellar vesicles (LUVs), 110 nm in diameter; small unilamellar vesicles (SUVs), 60 nm in diameter. The SUV is already highly curved. c,d) Ca^2+^‐dependent LDCV fusion with PM‐liposomes, either LUV or SUV. e) Dose‐response curves of Ca^2+^ on LDCV fusion with either LUV or SUV. Data are mean ± SEM (*n* = ≈5–10 independent experiments). Two‐way ANOVA with Bonferroni correction was applied to compare fusion with either LUV or SUV; ****, *p* < 0.0001. f,g) SV fusion with PM‐liposomes, either LUV or SUV. Mouse SVs are ≈45 nm in diameter. Data in (f,g) are mean ± SD (*n* = 6 independent experiments). Unpaired two‐tailed *t*‐test; ****, *p* < 0.0001.

To this end, we used different PM‐liposomes of different sizes: large unilamellar vesicles (LUV) with 110 nm diameter and small unilamellar vesicles (SUV) with 60 nm diameter (Experimental Section, Figure S5, Supporting Information). The average diameter of LDCVs is 150 nm, ranging from 100 to 300 nm.^[^ ^25c^ ^]^ We expected that the changes in local membrane curvature and tension induced by synaptotagmin‐1 would be minimized in small liposomes (**Figure** **5**a,b) because they are already highly curved.^[^ ^29b^ ^]^ Indeed, Ca^2+^‐dependent LDCV fusion was dramatically lower when SUVs were used compared to LUVs (Figure 5c–e). The curvature effect on vesicle fusion was reproduced when the concentration of liposomes and the ratio of vesicle to liposome were changed (Figure S4a–d, Supporting Information).

To further assess the effect of membrane curvature on vesicle fusion, we used SVs that have an average diameter of 45 nm.^[^ ^32^ ^]^ As expected, Ca^2+^‐dependent SV fusion with SUVs was completely impaired (Figure 5f,g), whereas basal SV fusion was already saturated and augmented (Figure 5g). Next, we replaced native SVs with small liposomes to confirm the curvature effect on vesicle fusion; small V‐liposomes (SUV) that incorporate full‐length VAMP‐2 and synaptotagmin‐1 fuse with small PM‐liposomes (SUV) either in the presence or absence of cholesterol (Figure S4e,f, Supporting Information). Indeed, no Ca^2+^‐dependent fusion was observed in the case of small V‐liposomes and small PM‐liposomes, whereas LUVs show Ca^2+^‐dependent liposome fusion (Figure 3d–f). Altogether, high membrane tension and curvature elevate basal fusion but decrease Ca^2+^‐dependent vesicle fusion, because highly curved membranes are likely to fuse without Ca^2+^ and show less change in membrane tension by membrane insertion of synaptotagmin‐1.

### 2.6 Cholesterol Strengthens Membrane Bending and Deformation

**Figure 6:**
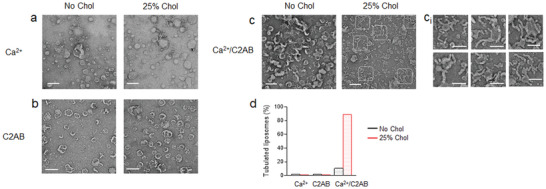
Cholesterol strengthens membrane deformation induced by the C2AB domain. TEM images of PM‐liposomes in the absence or presence of 25% Chol, incubated with either 100 µm Ca^2+^ (a), 2 µm C2AB domain (b), or Ca^2+^/C2AB domain (c). Scale, 200 nm. (c_i_) High magnification images of tubulated liposomes (white dotted lines in the right panel of c). Scale, 100 nm. d) Quantification of tubulated liposomes is presented as a percentage of total liposomes (*n* = 142, 188, 110, 184, 187, 475 from three independent experiments).

The C2AB domain of synaptotagmin‐1 has tubulation activity by membrane deformation and curvature generation.^[^ ^29^ ^]^ Next, we performed a tubulation assay using negative stain TEM to test that cholesterol regulates local membrane bending and deformation induced by synaptotagmin‐1 (**Figure** **6**). As expected, the Ca^2+^/C2AB domain induced tubulation by deforming the membrane in the presence of cholesterol, whereas either Ca^2+^ or the C2AB domain alone had no effect (Figure 6a–d), as correlating with the previous reports.^[^ ^29^ ^]^ Indeed, we observed no tubulation activity of the Ca^2+^/C2AB domain in the absence of cholesterol in PM‐liposomes (Figure 6c,d). These TEM data provide evidence that cholesterol is crucial for strengthening membrane bending and deformation caused by synaptotagmin‐1.

## 3 Discussion

Cholesterol regulates vesicle fusion as follows. First, it induces the clustering of SNARE proteins in the plasma membrane. This clustering could increase the efficiency of membrane fusion.^[^ ^24^ ^]^ Second, cholesterol causes negative membrane curvature that might facilitate and promote vesicle fusion;^[^ ^10^, ^21^, ^33^ ^]^ the negative curvature created by cholesterol can help bring two membranes closer together, making it easier for two membranes to fuse. Third, cholesterol contributes to vesicle fusion by stabilizing fusion pores;^[^ ^21^, ^31^, ^34^ ^]^ cholesterol might stabilize fusion pores by promoting negative membrane curvature.

**Figure 7:**
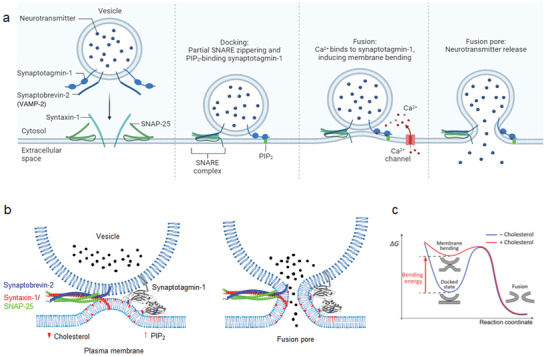
a) A schematic diagram summarizing synaptotagmin‐1‐induced membrane bending for Ca^2+^‐dependent vesicle fusion. Created with BioRender.com. b) PIP_2_ and cholesterol are critical for Ca^2+^‐dependent vesicle fusion; no Ca^2+^‐dependent vesicle occurs in the absence of PIP_2_ and cholesterol. Synaptotagmin‐1 mediates Ca^2+^‐dependent vesicle fusion by i) membrane insertion and ii) interacting with PIP_2_ through the polybasic patch, which induces the *trans*‐interaction of synaptotagmin‐1.^[^ ^25^, ^27^ ^]^ Cholesterol strengthens the plasma membrane deformation and bending caused by synaptotagmin‐1 insertion. Blue in synaptotagmin‐1, the polybasic patch interacting with PIP_2_; magenta, Ca^2+^. Membrane deformation is not stabilized without cholesterol, thus failing to trigger Ca^2+^‐dependent fusion. c) Energy landscapes of vesicle fusion by synaptotagmin‐1. Synaptotagmin‐1‐induced membrane bending enhanced by cholesterol generates significant membrane bending energy that can drive the membrane fusion by lowering the energy barriers to promote the fusion intermediate. Coarse‐grained molecular dynamics simulations predict the activation energies of ≈20–40 *k*_B_T for stalk and fusion pore formation,^[^ ^41^ ^]^ and experimental measurements show the activation energy of ≈30 *k*_B_T for vesicle fusion.^[^ ^42^ ^]^ The membrane bending energy generated by synaptotagmin‐1 might be used to overcome the energy barrier for vesicle fusion.^[^ ^35^ ^]^

The C2AB domains of synaptotagmin‐1 are inserted into membranes in a Ca^2+^‐dependent manner, and this membrane insertion into the lipid polar heads in one leaflet of the membrane leads to membrane curvature.^[^ ^35^ ^]^ Two hydrophobic residues in the Ca^2+^‐binding loops of synaptotagmin‐1 are partially inserted into the inner leaflet of the plasma membrane (≈10 Å deep).^[^ ^28^ ^]^ However, it remains unclear how membrane curvature induced by this weak and partial membrane insertion of synaptotagmin‐1 can be stabilized because the membrane is so highly dynamic and flexible that it can reform into its original shape. Cholesterol regulates the physical structure, fluidity, tension, and thickness of lipid membranes.^[^ ^21b^ ^]^ Molecular dynamics simulations^[^ ^36^ ^]^ and experimental mesoscale studies^[^ ^37^ ^]^ support that cholesterol increases the rigidity and decreases the fluidity of membranes, thereby stabilizing the membrane by increasing membrane stiffness, which makes the membrane more rigid. Our data provide evidence that synaptotagmin‐1 insertion deforms membranes and induces membrane curvature, which can be strengthened by cholesterol, thus lowering the energy barrier to trigger Ca^2+^‐dependent vesicle fusion (**Figure** **7**a–c).

How can membrane bending lower the energy barrier for fusion? i) The high membrane bending energy is likely to be released for fusion pore formation.^[^ ^38^ ^]^ For example, smaller vesicles,SUVs (Figure S4e,f, Supporting Information) and SVs (Figure 5f,g), have greater bending energy per unit of surface area and accordingly show robust basal fusion efficiency compared to large vesicles. The C2AB domain of synaptotagmin‐1 is inserted into the plasma membrane^[^ ^27^, ^28^ ^]^ and the membrane binding energy of the C2AB domain is ≈18 *k*_B_T^[^ ^39^ ^]^; the binding leads to local membrane bending and deformation.^[^ ^29^ ^]^ This curved plasma membrane has high bending energy, which can be released to drive fusion with vesicle membranes^[^ ^29^, ^38^ ^]^ (Figure 7b,c). ii) A mechanical force generated by membrane bending might help to bring the two membranes into close proximity,^[^ ^40^ ^]^ thus reducing the energy barrier for fusion (Figure 7c).

Cholesterol contributes to the formation of curved regions in the membrane for vesicle fusion,^[^ ^11^ ^]^ suggesting that membrane bending could accelerate fusion by reducing the energy barrier. The energy barrier for stalk formation and fusion pore prevents spontaneous fusion, and the activation energy represents the minimum amount of energy required for two membranes to fuse together.^[^ ^41^ ^]^ Coarse‐grained molecular dynamics simulations predict the activation energies of ≈20–40 *k*_B_T for stalk and fusion pore formation,^[^ ^41^ ^]^ and experimental measurements show the activation energy of ≈30 *k*_B_T for vesicle fusion.^[^ ^42^ ^]^ These data support that the energy barrier for fusion can be overcome by applying mechanical force such as membrane bending;^[^ ^35^ ^]^ this membrane bending energy might be used to overcome the energy barrier for vesicle fusion.

Here, our data provide a novel model that cholesterol strengthens local membrane bending and deformation induced by Ca^2+^‐dependent synaptotagmin‐1 insertion that generates membrane bending energy. Thus, high membrane bending energy strengthened by cholesterol lowers the energy barrier for fusion, thereby driving Ca^2+^‐dependent vesicle fusion (Figure 7a,b). Overall, membrane bending can lower the energy barrier to fusion by providing membrane bending energy and mechanical forces to trigger membrane fusion (Figure 7c).

Computational simulations also reproduce synaptotagmin‐1‐induced membrane bending and curvature.^[^ ^43^ ^]^ Simulations show that i) electrostatic interactions favor the *trans*‐interaction of synaptotagmin‐1 due to the higher negative charge density of PIP_2_ in the plasma membrane.^[^ ^43a^ ^]^ ii) The polybasic patch in the C2B domain induces membrane bending through interacting with PIP_2_ in a Ca^2+^‐dependent^[^ ^43b,c^ ^]^ and Ca^2+^‐independent manner.^[^ ^43a^ ^]^ iii) Synaptotagmin‐1 binding to the planar membranes generates higher membrane bending energy that drives vesicle fusion.^[^ ^43a^ ^]^ Simulations support our previous data that PIP_2_ is essential for triggering synaptotagmin‐1‐mediated Ca^2+^‐dependent vesicle fusion by inducing the *trans*‐interaction of synaptotagmin‐1 through the polybasic patch^[^ ^25^, ^27^ ^]^ (Figure 7a,b).

Holz and his colleagues reported pre‐fusion membrane curvature changes and plasma membrane deformations prior to vesicle fusion in chromaffin cells.^[^ ^44^ ^]^ Recently, Zuber's team observed membrane bending upon calcium influx before fusion occurs in synaptosomes.^[^ ^45^ ^]^ Using time‐resolved cryo‐electron tomography within 7 to 35 ms after diffusion of 52 mm KCl in synaptosomes, fusion initiation occurs by membrane curvature (“buckling”) of the plasma membrane, showing Ca^2+^‐dependent membrane bending before full fusion.^[^ ^45^ ^]^ This cryo‐electron tomography correlates with and strongly supports our model that the Ca^2+^‐bound C2AB domain of synaptotagmin‐1 induces membrane bending and curvature for vesicle fusion.

Our in vitro reconstitution of vesicle fusion has the advantage of using purified native vesicles, LDCVs and SVs, and reproduces the physiological cholesterol effect on vesicle fusion, whereas V‐liposomes fail to reproduce vesicle fusion in a physiological context (Figure 3d–f). V‐liposomes are independent of cholesterol for Ca^2+^‐dependent fusion, in contrast to native vesicles (Figures 2 and 3). V‐liposomes might have some limitations in fully replacing native vesicles for a fusion assay.

What distinguishes native vesicles from V‐liposomes for a fusion assay? Native vesicles differ from V‐liposomes in lipid composition, protein density, contents diversity, and physical properties; therefore, the structural integrity of native vesicles enables to mimic endogenous vesicle fusion in the in vitro reconstitution. Regarding the physical and structural integrity, we observed that both LDCVs and SVs remain stable and functionally active even after ≈3–5 times freeze‐thaw cycles.^[^ ^25a,c^ ^]^ Despite ≈3–5 times snap freeze‐thaw cycles, biophysical and biochemical properties of purified LDCVs are preserved; i) the structure and size distribution of LDCVs are normal, ii) SNARE‐dependent fusion of LDCVs is reproduced, and iii) acidification of LDCVs is fully functional,^[^ ^25c^ ^]^ suggesting that the membrane stability and rigidity of native vesicles after freeze‐thaw cycles remain stable and functional. In contrast, proteoliposomes become immediately disrupted and destructed by freezing regardless of cholesterol in liposomes, thus these disrupted liposomes fail to undergo fusion. Liposomes should not be frozen, because the freezing process fractures or ruptures liposomes, thus leading to the destruction of liposome membranes.

However, vesicular proteins, such as scaffold proteins and granins, could stabilize vesicle structure and regulate membrane rigidity.^[^ ^46^ ^]^ Native vesicles have high membrane rigidity compared to V‐liposomes, and native vesicles seem to require higher membrane bending energy than V‐liposomes do, in order to overcome the barrier for fusion due to membrane rigidity. V‐liposomes might have a low energy barrier for Ca^2+^‐dependent fusion, and membrane bending is not necessary to overcome this energy barrier; therefore, cholesterol is not likely required for liposome‐liposome fusion. The structural integrity of native vesicles can be advantageous for the in vitro reconstitution of vesicle fusion to reproduce physiological exocytosis, but the causes of the differences between native vesicles and liposomes for the fusion efficiency in a physiological context remain topics for further study.

The molecular mechanisms of synaptotagmin‐1 to trigger Ca^2+^‐dependent fusion remain controversial; at least six different competing models have been proposed.^[^ ^17^ ^]^ Synaptotagmin‐1 mediates Ca^2+^‐dependent fusion by the electrostatic interaction, so several different synaptotagmin‐1 models have been proposed, depending on different ionic environments.^[^ ^25b^ ^]^ Here, we confirmed the cholesterol effect on vesicle fusion in a physiological ionic environment, that is, normal ionic strength with Mg^2+^/ATP. Both the C2A and C2B domains of synaptotagmin‐1 are inserted into the plasma membrane (Figure 4i) without interacting with SNARE proteins,^[^ ^25b^ ^]^ and therefore lead to bending and deformation of the plasma membrane (Figure 6). Ca^2+^ fails to trigger fusion in the absence of cholesterol despite the proteins, both SNARE assembly and membrane binding of synaptotagmin‐1, being fully active and functional (Figures 2 and 4). Cholesterol has a critical function in Ca^2+^‐dependent fusion, as an essential lipid regulator through strengthening membrane deformation and curvature caused by synaptotagmin‐1.

Age‐related cholesterol reduction is linked to reduced synaptic activity, and defects in synaptic transmission by cholesterol deficiency could result in neurodegeneration.^[^ ^3^ ^]^ Our data explain the molecular mechanisms of how cholesterol contributes to synaptic transmission and neuronal function and may pave the way for the development of studies to explore the treatment of neurodegenerative and neurodevelopmental disorders by optimizing cholesterol levels in the plasma membrane.

## 4 Experimental Section

### Purification of Large Dense‐Core Vesicles and Synaptic Vesicles

LDCVs, also known as chromaffin granules, were purified from bovine adrenal medullae by using a continuous sucrose gradient and resuspended with fusion buffer containing 120 mm K‐glutamate, 20 mm K‐acetate, and 20 mm HEPES.KOH, pH 7.4, as elsewhere.^[^ ^25c^ ^]^ SV from mouse brains was purified as described elsewhere.^[^ ^47^ ^]^ Briefly, mice brains were homogenized in a homogenization buffer supplemented with protease inhibitors, using a glass‐Teflon homogenizer. The homogenate was centrifuged for 10 min at 1000 g and the resulting supernatant was further centrifuged for 15 min at 15 000 g. The synaptosome pellet was lysed by adding ice‐cold water, followed by centrifugation for 25 min at 48 000 g. The resulting supernatant was overlaid onto a 0.7 m sucrose cushion and centrifuged for 1 h at 133 000 g. The pellet was resuspended in a fusion buffer (120 mm K‐glutamate, 20 mm K‐acetate, 20 mm HEPES.KOH, pH 7.4).

### Protein Purification

All SNARE and the C2AB domain of synaptotagmin‐1 constructs based on *Rattus norvegicus* sequences were expressed in *Escherichia coli* strain BL21 (DE3) and purified by nickel–nitrilotriacetic acid (Ni^2+^‐NTA) affinity chromatography followed by ion‐exchange chromatography as described earlier.^[^ ^25a,b^ ^]^ The stabilized Q‐SNARE complex consisting of syntaxin‐1A (aa 183–288) and SNAP‐25A (no cysteine, cysteines replaced by alanines) in a 1:1 ratio by the C‐terminal VAMP‐2 fragment (aa 49–96) was purified as described earlier.^[^ ^26^ ^]^ The binary Q‐SNARE complex containing the full‐length syntaxin‐1A (1‐288) and SNAP‐25A (no cysteine, cysteines replaced by alanines) was expressed using co‐transformation.^[^ ^25a^ ^]^ The full‐length VAMP‐2, soluble cytoplasmic region of VAMP‐2 (VAMP‐2_1‐96_), full‐length synaptotagmin‐1, C2AB domain of synaptotagmin‐1 (aa 97–421), C2A domain (aa 96–262), C2B domain (aa 248–421), and C2ab mutant (D178A, D230A, D232A, D309A, D363A, D365A) were purified as described previously.^[^ ^48^ ^]^ The stabilized Q‐SNARE complex and the syntaxin‐1A/SNAP‐25A binary SNARE complex were purified by Ni^2+^‐NTA affinity chromatography followed by ion‐exchange chromatography in the presence of 50 mm *n*‐octyl‐*β*‐D‐glucoside (OG).^[^ ^25a^ ^]^ The point mutated C2AB domain (S342C) was labeled with Alexa Fluor 488 C5 maleimide (C2AB^A488^).^[^ ^48^ ^]^ Protein structures were visualized with PyMOL; PDB 1BYN for the C2A domain, 1K5W for the C2B domain, and 3IPD for the SNARE complex.

### Lipid Composition of Liposomes

Lipid composition (molar percentages) of PM‐liposomes that contain the Q‐SNARE complex consisted of 45% PC (l‐*α*‐phosphatidylcholine, Cat. 840 055), 15% PE (l‐*α*‐phosphatidylethanolamine, Cat. 840 026), 10% PS (l‐*α*‐phosphatidylserine, Cat. 840 032), 25% Chol (cholesterol, Cat. 700 000), 4% PI (l‐*α*‐phosphatidylinositol, Cat. 840 042), and 1% PI(4,5)P_2_ (Cat. 840 046). When cholesterol was excluded (0% Chol), PC contents were accordingly adjusted. In case of changing PI(4,5)P_2_ concentration, PI contents were accordingly adjusted. VAMP‐2/synaptotagmin‐1‐containing V‐liposomes were composed of 55% PC, 20% PE, 15% PS, and 10% Chol. For FRET‐based lipid‐mixing assays, 1.5% 1,2‐dioleoyl‐sn‐glycero‐3‐phosphoethanolamine‐*N*‐(7‐nitrobenz‐2‐oxa‐1,3‐diazol‐4‐yl (NBD‐DOPE) and 1.5% 1,2‐dioleoyl‐sn‐glycero‐3‐phosphoethanolamine‐N‐lissamine rhodamine B sulfonyl ammonium salt (Rhodamine‐DOPE) were incorporated in PM‐liposomes (accordingly 12% unlabeled PE) as a donor and an acceptor dye, respectively. For FRET measurement using C2AB^A488^, 1.5% Rhodamine‐DOPE was included in PM‐liposomes (protein‐free). In the case of FRET for tryptophan, 5% *N*‐(5‐dimethylaminonaphthalene‐1‐dulfonyl)‐1,2‐dihexadecanoyl‐sn‐glycero‐3‐phosphoethanolamine, triethylammonium salt (dansyl‐DHPE) was incorporated in PM‐liposomes (protein‐free). All lipids were from Avanti Polar lipids except dansyl‐DHPE (Invitrogen).

### Preparation of Proteoliposomes

Incorporation of the stabilized and binary Q‐SNARE complex into LUVs (110 nm in diameter) was achieved by OG‐mediated reconstitution, called the direct method, that is, incorporation of proteins into preformed liposomes.^[^ ^25a,b^ ^]^ Briefly, lipids dissolved in chloroform were mixed according to lipid composition. The solvent was removed using a rotary evaporator (which generated lipid film on a glass flask), then lipids were resuspended in 0.5 mL buffer containing 150 mm KCl and 20 mm HEPES/KOH pH 7.4. After sonication on ice , multilamellar vesicles were extruded using polycarbonate membranes of pore size 100 nm (Avanti Polar lipids) to give uniformly‐distributed LUVs with an average diameter of 110 nm (Figure S5, Supporting Information). After the preformed LUVs had been prepared, SNARE proteins or the full‐length VAMP‐2/synaptotagmin‐1 were incorporated into them using OG, a mild non‐ionic detergent, then the OG was removed by dialysis overnight in 1 L buffer containing 150 mm KCl and 20 mm HEPES/KOH pH 7.4 together with 2 g SM‐2 adsorbent beads. Unless otherwise stated, both V‐liposomes and PM‐liposomes were LUVs.

To make SUVs using the direct method, 110‐nm LUVs produced as described above were extruded through polycarbonate membranes with 50‐nm pore size (yielding SUV that had an average diameter of 60 nm, Figure S5, Supporting Information). After preparing preformed SUVs, protein incorporation was completed by OG as described for LUV. The size distribution of proteoliposomes was determined using dynamic light scattering (DLS) (Figure S5, Supporting Information). Proteoliposomes had a protein‐to‐lipid molar ratio of 1:500 (n/n).

### Vesicle Fusion Assay

A FRET‐based lipid‐mixing assay was applied to monitor vesicle fusion in vitro.^[^ ^25a,b^ ^]^ LDCV or SV fusion reactions were performed at 37 °C in 1 mL fusion buffer containing 120 mm K‐glutamate, 20 mm K‐acetate, 20 mm HEPES‐KOH (pH 7.4), 1 mm MgCl_2_, and 3 mm ATP. ATP should be made freshly before experiments because ATP is easily destroyed by freezing and thawing. Free Ca^2+^ concentration in the presence of ATP and Mg^2+^ was calibrated using the MaxChelator simulation program. The fluorescence dequenching signal was measured using Fluoromax (Horiba Jobin Yvon) with wavelengths of 460 nm for excitation and 538 nm for emission. Fluorescence values were normalized as a percentage of maximum donor fluorescence (total fluorescence) after the addition of 0.1% Triton X‐100 at the end of experiments.

### Fluorescence Resonance Energy Transfer

The C2AB domain of synaptotagmin‐1 (30 nm, S342C) was labeled with Alexa Fluor 488, a donor dye. C2AB fragment was engineered to contain a single Cys residue (S342C) and labeled with Alexa Fluor 488. 1.5% Rhodamine‐DOPE (Rho‐PE), incorporated in PM‐liposomes (protein‐free), was used as an acceptor. Unless otherwise stated, liposomes were LUVs prepared by the direct method. The donor fluorescence signal was measured at 37 °C using Fluoromax (Horiba Jobin Yvon) with wavelengths of 488 nm for excitation and 516 nm for emission in 1 mL buffer containing 120 mm K‐glutamate, 20 mm K‐acetate, 20 mm HEPES‐KOH (pH 7.4), 1 mm MgCl_2_, and 3 mm ATP. FRET was normalized as net changes of donor fluorescence intensity and C2AB binding was presented as a percentage of maximum C2AB binding induced by 1 mm Ca^2+^. In Figure S3, Supporting Information, FRET was normalized as F/F_0_, where F_0_ represented the initial value of the donor fluorescence intensity.

Liposome binding of the C2AB domain was also monitored using the tryptophan‐dansyl FRET pair as a donor–acceptor dye in which dansyl‐DHPE incorporated in liposomes led to quenching of fluorescence emitted from tryptophan of the C2AB domain.^[^ ^49^ ^]^ Then1 *µ* m C2AB, 3 *µ* m C2A, or 3 *µ* m C2B was incubated with V‐liposomes (protein‐free). The donor fluorescence signal was measured at 37 °C using Fluoromax (Horiba Jobin Yvon) with wavelengths of 295 nm for excitation and 350 nm for emission in 1 mL buffer containing 120 mm K‐glutamate, 20 mm K‐acetate, 20 mm HEPES‐KOH (pH 7.4), 1 mm MgCl_2_, and 3 mm ATP. FRET monitoring C2AB binding was normalized as a percentage of (F_0_‐F)/F_0_, where F0 represents the initial value of the donor fluorescence intensity

### Fluorescence Anisotropy Measurements

The C2AB fragment (40 nm, S342C) were labeled with Alexa Fluor 488.^[^ ^48^ ^]^ Anisotropy was measured at 37 °C in 1 mL of buffer containing 120 mm K‐glutamate, 20 mm K‐acetate, 20 mm HEPES‐KOH (pH 7.4), 1 mm MgCl_2_, and 3 mm ATP. The excitation wavelength was 495 nm, and the emission was measured at 520 nm. F0The lipid composition of PM‐liposomes (protein‐free) was identical to those used in a fusion assay except for labeled PE (45% PC, 15% PE, 10% PS, 25% Chol, 4% PI, and 1% PIP_2_). In the case of 0% Chol, PC contents were adjusted accordingly (70% PC).

### Ternary SNARE Complex Formation Assay

TeNT degraded free VAMP‐2 whereas VAMP‐2, assembled in the ternary SNARE complex, becomes resistant to TeNT.^[^ ^25a^ ^]^ After incubation of LDCVs with PM‐liposomes (0% or 25% Chol) containing the stabilized Q‐SNARE complex for 20 min at 37 °C without Ca^2+^. Then, the sample was subjected to TeNT treatment (200 nm, 30 min, 37 °C), and then boiled for 5 min at 95 °C and analyzed by immunoblotting with antibody against VAMP‐2 (clone number 69.1, Synaptic Systems (Göttingen, Germany)).

### Preparation of Bovine Chromaffin Cells

Chromaffin cells were isolated from the bovine adrenal gland medulla by two‐step collagenase digestion as previously described.^[^ ^50^ ^]^ The cells were grown on poly‐_D_‐lysine‐coated glass coverslips in Dulbecco's modified Eagle medium/F‐12 (Invitrogen, CA) containing 10% fetal bovine serum (Hyclone Laboratories, UT) and 1% antibiotics (Invitrogen, CA).

### Amperometric Measurement

Recordings of LDCV exocytosis from chromaffin cells were performed as described previously.^[^ ^50^ ^]^ Carbon‐fiber electrodes were fabricated with 8 µm diameter carbon fibers and back‐filled with 3 m KCl. The amperometric current, generated by the oxidation of catecholamine, was measured using an axopatch 200B amplifier (Axon Instruments Inc., CA), which was operated in voltage‐clamp mode at a holding potential of +650 mV. Amperometric signals were low‐pass filtered at 1 kHz and sampled at 500 Hz. For data acquisition and analysis, pCLAMP 11 software (Axon Instruments) was used. The area of amperometric current represented the total amount of released catecholamine. The amperometric current generated by repetitive stimulation was individually integrated. Relative exocytosis is presented as a percentage of the first DMPP‐induced total catecholamine release.Note that only one DMPP stimulation was applied before treatment to avoid any potentiation effect, because chromaffin cells show activity‐dependent potentiation of LDCV exocytosis by repetitive stimulation.^[^ ^50^ ^]^

### Transmission Electron Microscopy

Chromaffin cells, grown on Vitrogen collagen matrix (Cohesion, Palo Alto, CA), were washed out with Locke's solution containing 157.4 mm NaCl, 5.6 mm KCl, 2.2 mm CaCl_2_, 1.2 mm MgCl_2_, 5.6 mm d‐glucose, 5 mm HEPES, and 3.6 mm NaHCO_3_, pH 7.4 titrated by NaOH. As described previously,^[^ ^50^ ^]^ cells were fixed with 2% paraformaldehyde and 2% glutaraldehyde in 0.05 m sodium cacodylate buffer at pH 7.4 for 20 min at room temperature. Cells were post‐fixed with 0.5% osmium tetroxide in 0.05 m sodium cacodylate buffer at pH 7.4 for 30 min at room temperature. Cells were further dehydrated in graded ethanol solutions and embedded in LR White resin (London Resin Co., Berkshire, UK). Silver‐gold thin sections were stained with uranyl acetate and lead citrate. The thin sections were examined under JEOL 1200 EX2 TEM at 80 kV.

For liposomes, 5 µL of samples were deposited on carbon‐coated 400‐mesh copper grids (CF400‐CU, Electron Microscopy Sciences). Grids were stained with uranyl acetate for negative staining and embedded in methylcellulose‐uranyl acetate. Liposomes were visualized at 80 kV in Talos F200C TEM (Thermo Fisher Scientific). The images were acquired using a bottom‐mounted CETA camera.

### Liposome co‐Flotation Assay

Liposomes float up through the gradient due to their buoyancy and free proteins remain at the bottom of the gradient, whereas proteins incorporated in liposomes co‐float to the buoyant density of the liposomes.^[^ ^51^ ^]^ First, 30 µL of liposomes that incorporate the stabilized Q‐SNARE complex were mixed with Nycodenz (Axis Shield, 80%, 30 µL), and a second Nycodenz layer (30%, 40 µL) was gently applied followed by another layer of buffer (40 µL). The density gradient was centrifuged using a Beckman TL‐100 ultracentrifuge (TLS55 rotor, 100 000 g, 4 °C, 1 h). The 20‐µL aliquots were carefully taken from the top of the gradient and analyzed by Coomassie blue staining.

### Statistical Analysis

Data analysis was performed using OriginPro 2019 software (OriginLab Corporation, Northampton, MA, USA) and GraphPad Prism 9 (GraphPad Software, San Diego, CA, USA). Data are means ± standard deviation (SD) or standard error of mean (SEM). One‐way ANOVA test with Bonferroni correction was used to determine any statistically significant differences between three or more independent groups. Two‐way ANOVA test with Bonferroni correction was applied to compare two dose‐response curves. Unpaired two‐tailed *t*‐test was used to estimate the statistical significance between the two groups. Probabilities of *p* < 0.05 were considered significant. Dose‐response curves were fitted using four‐parameter logistic equations (GraphPad Prism) to calculate EC_50_.

## Conflict of Interest

The authors declare no conflict of interest.

## Author Contributions

H.Y.A.M. and Y.P. contributed equally to this work. Y.P., H.Y.A.M., and K.C.S. purified vesicles and performed experiments. J.P. and S.M. conducted and analyzed TEM for liposomes and S.J.K. carried out TEM for chromaffin cells. J.K.R. did modeling. Y.P. conceived the idea, designed the study, collected and analyzed data. Y.P. wrote the manuscript and all authors read and provided their comments.

## Supporting information

Supporting Information

Supporting Information

## Data Availability

The data that support the findings of this study are available in the supplementary material of this article.
