# Supplementary material for: Requirement of Cholesterol for Calcium‐Dependent Vesicle Fusion by Strengthening Synaptotagmin‐1‐Induced Membrane Bending
Source: Adv Sci (Weinh). 2023 Apr 14;10(15):2206823. doi: 10.1002/advs.202206823 (PMC10214243; doi:10.1002/advs.202206823)

ADVANCED SCIENCE
Open Access

Supporting Information

for *Adv. Sci.*, DOI 10.1002/advs.202206823

Requirement of Cholesterol for Calcium-Dependent Vesicle Fusion by Strengthening Synaptotagmin-1-Induced Membrane Bending

*Houda Yasmine Ali Moussa, Kyung Chul Shin, Janarthanan Ponraj, Soo Jin Kim, Je-Kyung Ryu, Said Mansour and Yongsoo Park**

Supporting Information (SI)

Supplementary Figure 1

Incorporation of the Q-SNARE complex in PM-liposomes. (**a-c**) Incorporation of the Q-SNARE complex in liposomes in the presence or absence of cholesterol was tested using a flotation assay. (**a**) Schematic diagram showing that liposomes float up through the gradient due to their buoyancy, whereas free proteins remain in the bottom of the gradient. (**b,c**) SNARE proteins stained by Coomassie blue dyes. The Q-SNARE proteins SNAP-25A (no cysteine, cysteines are replaced by alanines) and syntaxin-1A (aa 183–288) in a 1:1 ratio by the C-terminal VAMP-2 fragment (aa 49–96), are incorporated in liposomes that contained either 25% Chol (**b**) or no Chol (**c**).

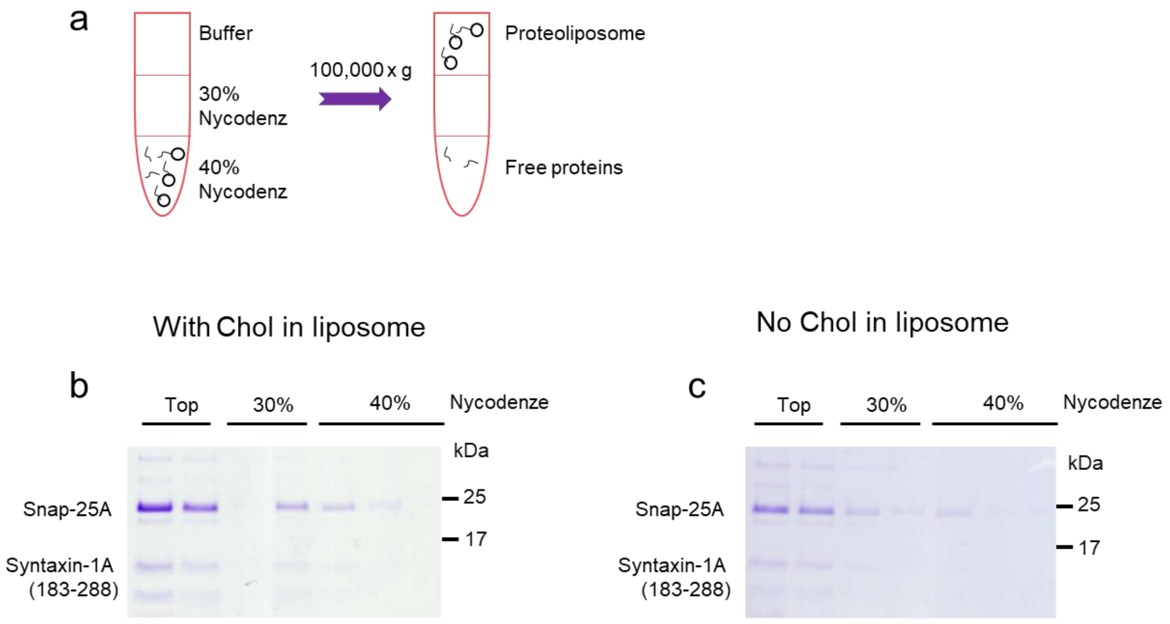

Supplementary Figure 2

Cholesterol requirement for Ca^{2+}-evoked LDCV fusion. (**a**) No Ca^{2+}-dependent LDCV fusion was observed when cholesterol was absent in PM-liposomes (0% Chol). The Q-SNARE complex consisting of the full-length syntaxin-1A (1-288) and SNAP-25A (no cysteine, cysteines are replaced by alanines) was incorporated in PM-liposomes. The binary Q-SNARE complex shows slow fusion rate and relatively low fusion activity, as expected[25a, 26]. Similar results were obtained from four independent trials.

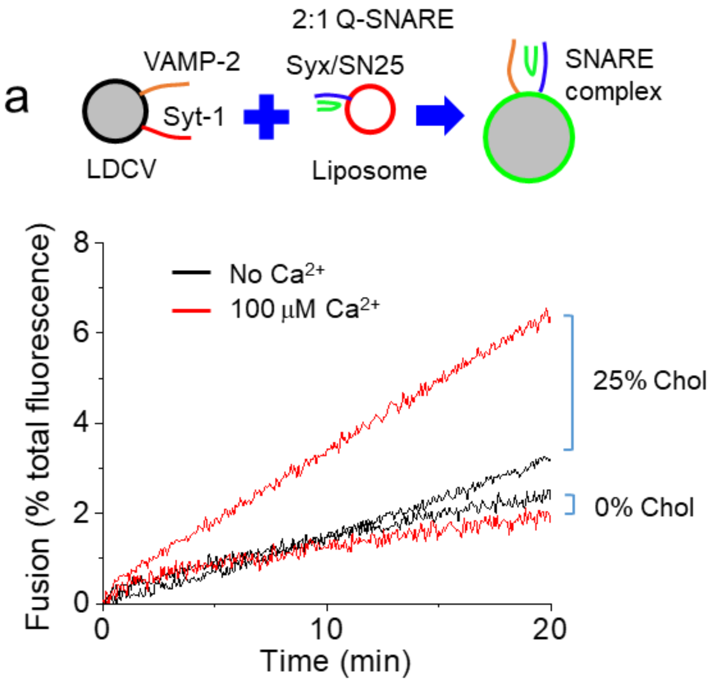

Supplementary Figure 3

Monitoring C2AB binding to liposomes using FRET measurement. (**a**) A schematic diagram of C2AB domain binding to PM-liposomes. Membrane binding of the C2AB domain of synaptotagmin-1 was monitored using FRET in which the C2AB domain (Syt-$1_{97\text{-}421}$) was labelled with Alexa Fluor 488 at S342C (green dots) as a donor, and PM-liposomes (Lip.) were labelled with Rhodamine (Rho)-PE (red) as an acceptor (**Online Methods**). Lipid composition of PM-liposomes: 45% PC, 13.5% PE, 10% PS, 25% Chol, 4% PI, 1% PIP_2, and 1.5% Rho-PE. When PIP_2 was changed, PI contents were adjusted accordingly. (**b**) PIP_2 contents were increased to 5% in PM-liposomes, and C2AB binding was saturated with 0.5% PIP_2 in PM-liposomes. 1 mM free Ca^{2+} induced maximum C2AB binding in the presence of 1 mM $MgCl_2$/3 mM ATP.

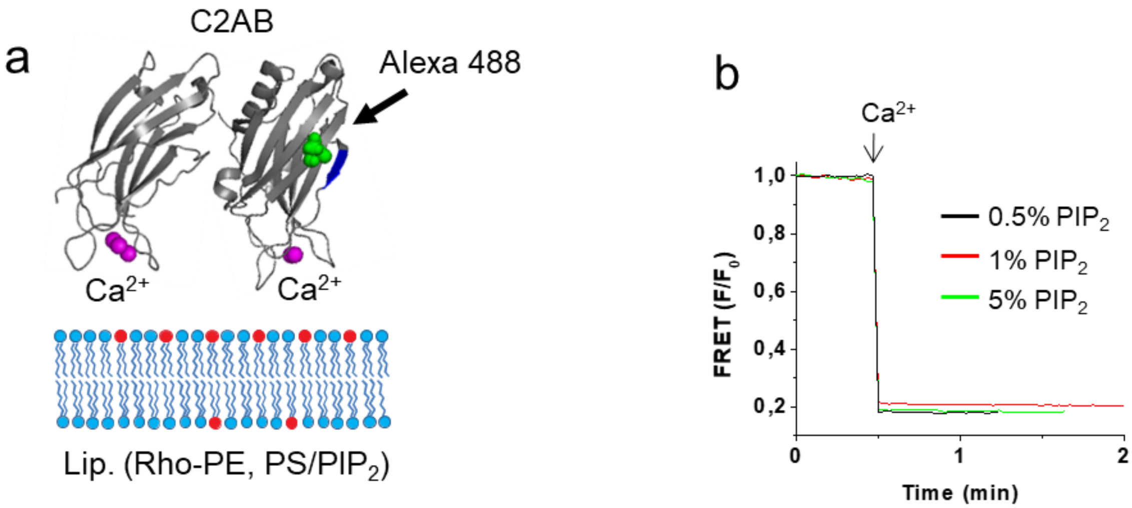

Supplementary Figure 4

Ca^{2+}-dependent vesicle fusion depends on liposome membrane curvature. Large unilamellar vesicles (LUVs), 110 nm in diameter; small unilamellar vesicles (SUVs), 60 nm in diameter. A lipid-mixing assay to monitor Ca^{2+}-dependent LDCV fusion with PM-liposomes, either LUV or SUV. Equal amount of LDCVs (5 µL) was incubated to induce fusion with either 5 µL (**a,b**) or 20 µL (**c,d**) LUV or SUV in 1 mL fusion buffer; 120 mM K-glutamate, 20 mM K-acetate, 20 mM HEPES-KOH (pH 7.4), 1 mM $MgCl_2$, and 3 mM ATP. 100 µM free Ca^{2+} in the presence of 1 mM $MgCl_2$/3 mM ATP. (**e,f**) Both V-liposomes and PM-liposomes are SUVs. V-liposomes incorporating the full-length synaptotagmin-1 and VAMP-2 were incubated with PM-liposomes that contained either 25% (**e**) or 0% (**f**) Chol. Lipid composition of PM-liposomes and V-liposomes is described in **Figure 2** and **Figure 3**, respectively. Similar results were obtained from three independent trials.

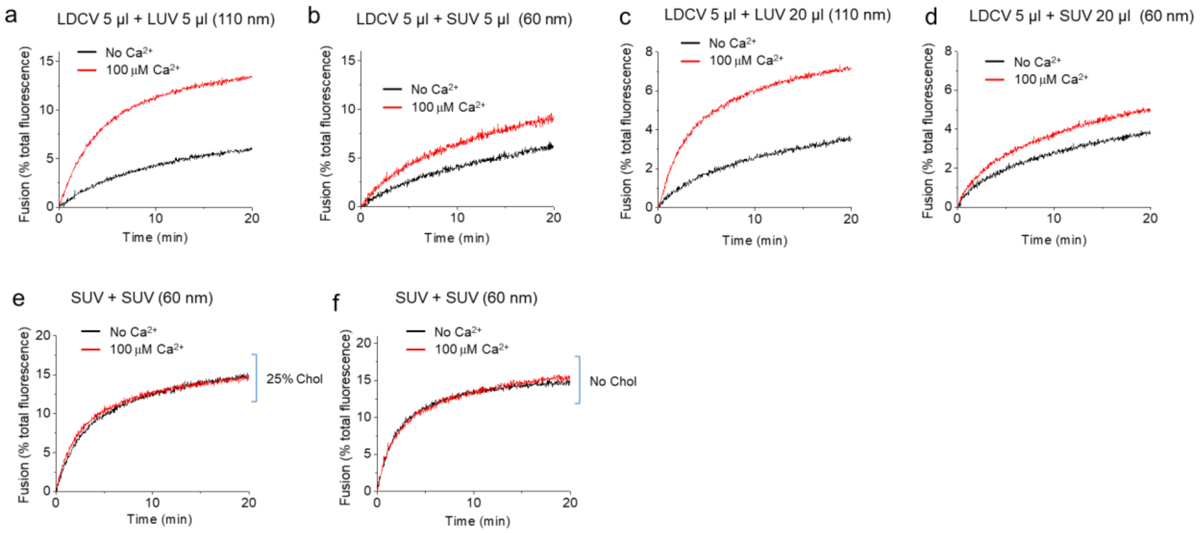

Supplementary Figure 5

The size distribution of proteoliposomes. The size distribution of proteoliposomes was determined using dynamic light scattering (DLS). LUVs (110 nm in diameter) and SUVs (60 nm in diameter) were prepared by extrusion (**Online Methods**).

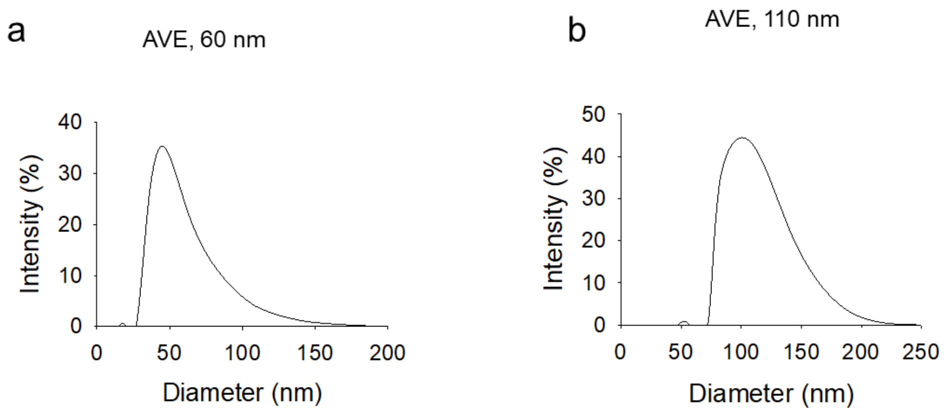

Supplement: Supplementary file 1 — Supporting Information [file ADVS-10-2206823-s001.pdf]
